# Cognitive behaviour therapy response and dropout rate across purging and nonpurging bulimia nervosa and binge eating disorder: DSM-5 implications

**DOI:** 10.1186/1471-244X-13-285

**Published:** 2013-11-07

**Authors:** Zaida Agüera, Nadine Riesco, Susana Jiménez-Murcia, Mohammed Anisul Islam, Roser Granero, Enrique Vicente, Eva Peñas-Lledó, Jon Arcelus, Isabel Sánchez, Jose Manuel Menchon, Fernando Fernández-Aranda

**Affiliations:** 1 CIBER Fisiopatología de la Obesidad y Nutrición (CIBERobn), Instituto Salud Carlos III, Madrid, Spain; 2 Department of Psychiatry, University Hospital of Bellvitge-IDIBELL, Barcelona, Spain; 3 Department of Clinical Sciences, School of Medicine, University of Barcelona, Barcelona, Spain; 4 Departament de Psicobiologia i Metodologia, Universitat Autònoma de Barcelona, Barcelona, Spain; 5 CICAB Clinical Research Centre, Extremadura University Hospital and Medical School, Badajoz, Spain; 6 Leicester Eating Disorder Service, Brandon Mental Health Unit, Leicester General Hospital, Leicester, UK; 7 CIBER Salud Mental (CIBERSAM), Instituto Salud Carlos III, Barcelona, Spain

**Keywords:** Binge eating disorder (BED), Bulimia nervosa (BN), Cognitive-behavioural therapy (CBT), Classification, DSM-5

## Abstract

**Background:**

With the imminent publication of the new edition of the Diagnostic and Statistical Manual of Mental Disorders (DSM-5), there has been a growing interest in the study of the boundaries across the three bulimic spectrum syndromes [bulimia nervosa-purging type (BN-P), bulimia nervosa-non purging type (BN-NP) and binge eating disorder (BED)]. Therefore, the aims of this study were to determine differences in treatment response and dropout rates following Cognitive Behavioural Therapy (CBT) across the three bulimic-spectrum syndromes.

**Method:**

The sample comprised of 454 females (87 BED, 327 BN-P and 40 BN-NP) diagnosed according to DSM-IV-TR criteria who were treated with 22 weekly outpatient sessions of group CBT therapy. Patients were assessed before and after treatment using a food and binging/purging diary and some clinical questionnaires in the field of ED. “Full remission” was defined as total absence of binging and purging (laxatives and/or vomiting) behaviors and psychological improvement for at least 4 (consecutive).

**Results:**

Full remission rate was found to be significantly higher in BED (69.5%) than in both BN-P (p < 0.005) and BN-NP (p < 0.001), which presented no significant differences between them (30.9% and 35.5%). The rate of dropout from group CBT was also higher in BED (33.7%) than in BN-P (p < 0.001) and BN-NP (p < 0.05), which were similar (15.4% and 12.8%, respectively).

**Conclusions:**

Results suggest that purging and non-purging BN have similar treatment response and dropping out rates, whereas BED appears as a separate diagnosis with better outcome for those who complete treatment. The results support the proposed new DSM-5 classification

## Background

With the imminent publication of the new edition of the Diagnostic and Statistical Manual of Mental Disorders (DSM-5) [1], the validity of the current diagnostic criteria for Eating Disorders (ED), in particular the boundaries between the three bulimic-spectrum syndromes (Bulimia Nervosa Purging, BN-P; Bulimia Nervosa Non-Purging, BN-NP; and Binge Eating Disorder, BED) [2-4], mostly characterized by the presence of binge episodes as main symptom, has been a topic of ongoing debate [5-7]. This forthcoming edition recommends the recognition of BED as a freestanding diagnosis and the maintenance of the purging and non-purging subtypes of BN in the same category. Although this proposal is based on some scientific/clinical evidence reviewed below, there is still little agreement about best to define the subtyping groups [6].

Several studies have investigated clinical and non-clinical differences between the three bulimic groups. However, no consensus appears to exist in the literature comparing BED and BN. While some studies have shown no differences between the three bulimic syndromes with regard to eating disorders psychopathology [8,9], psychiatric co-morbidity [10] or personality factors [11], studies comparing BED vs. BN have shown differences in the levels of obesity [3,12,13], food restriction [5,14], co-morbidity [15], and prognosis [16,17] between them. Similarly, studies have also identified higher rates of psychiatric co-morbidity and psychopathology in BN-P when comparing to BN-NP [3,18,19]. In agreement with the observed differences between subtypes, some authors have proposed a continuum of clinical severity across the three bulimic diagnostic subgroups, with BN-P at the top end of severity and BED at the bottom end [3,17].

One of the main issues involved in the revisions for DSM-5 is the predictive validity of diagnostic criteria with regard to outcome [20]. It is surprising that, in spite of a large number of studies investigating clinical differences between the three diagnostic groups, no studies have examined differences in treatment response between them, particularly for Cognitive Behavioural Therapy (CBT) whose effectiveness for the treatment of bulimic disorders has been demonstrated in numerous randomized controlled trials [21-25]. Hay and Fairburn [26], in a longitudinal two-stage design general population study, assessed and compared the stability of bulimic disorders one year after recruitment, but they did not take treatment into account.

Furthermore, in the current literature there is also lack of information about treatment dropout across the three bulimic syndromes. In this regard, only Graham and Walton [27] found higher rates of dropout in BN when compared to BED patients using CD-Rom CBT. These authors postulated that BN presented with more severe eating disorders symptomatology than BED, which contributed to a higher rate of treatment dropout rate in this group of patients (BN).

Therefore, to our knowledge, this is the first study assessing response to treatment and dropout rates between the three disorders (BN-P, BN-NP and BED) after a group CBT treatment, which may contribute to advancements in the debate about whether the three diagnoses are separate domains or not.

### Aims of the study

The aims of the present study are twofold: 1) To determine the rate of response to CBT across BN-P, BN-NP and BED and 2) To describe the differences in the rate of dropout across the three diagnostic groups.

We hypothesized the finding of dimensional differences across the three diagnoses with BN-P representing the most severe and BED the least. Therefore, we expect the BED group to exhibit the most clinical improvement, followed by the BN-NP group and the BN-P group to show the least recovery. Likewise, we expect to find the highest risk of dropout in BN-P patients.

## Methods

### Participants

Every female patient over the age of 18 years who was diagnosed with BNP, BN-NP or BED according to DSM-IV-TR [28] criteria was invited to participate in this study. All participants were consecutively admitted for group-based outpatient treatment with no other psychotherapy at the time at the Eating Disorders Unit (University Hospital of Bellvitge), between 1998 and 2009. Patients were excluded if they presented with severe co-morbid psychopathological symptoms (e.g., suicidal attempts) requiring individual and/or inpatient therapy.

### Assessment

For the assessment, commonly applied questionnaires in the field of EDs, comprising the Eating Disorders Inventory-2 (EDI-2) [29] and the Symptom Checklist-Revised (SCL-90-R) [30] were employed.

#### Eating disorders inventory-2 (EDI-2) [29]

This is a reliable and valid 91-item multidimensional self-report questionnaire that assesses different cognitive and behavioural characteristics, which are typical for EDs. The EDI-2 retains the 64 items grouped into eight scales: Drive for Thinness (DT), Bulimia (B), Body Dissatisfaction (BD), Ineffectiveness (I), Perfectionism (P), Interpersonal Distrust (ID), Interoceptive Awareness (IA), Maturity Fears (MF) of the EDI and adds 27 new items into three provisional scales: Asceticism (A), Impulse Regulation (IR), and Social Insecurity (SI). All of these scales are answered on a 6-point Likert scale, and provide standardized subscale scores. When this instrument was validated in a Spanish population [31], a mean internal consistency of 0.63 (coefficient alpha) was found.

#### Symptom checklist- revised (SCL-90-R) [30]

In order to evaluate a broad range of psychological problems and symptoms of psychopathology, the SCL-90-R was employed. This test contains 90 items and helps measure 9 primary symptom dimensions, which are: 1) Somatization; 2) Obsession-Compulsion; 3) Interpersonal Sensitivity; 4) Depression; 5) Anxiety; 6) Hostility, 7) Phobic Anxiety; 8) Paranoid Ideation and 9) Psychoticism. In addition, it includes three global indices, which are a global severity index (GSI), designed to measure overall psychological distress; a positive symptom distress index (PSDI), designed to measure the intensity of symptoms as well as a positive symptom total (PST), which measures self-reported symptoms. The Global Severity Index can be used as a summary of the test. This scale has been validated in a Spanish population [32], obtaining a mean internal consistency of 0.75 (Coefficient alpha).

### Procedure

Experienced psychologists and psychiatrists diagnosed all participants according to the DSM-IV-TR [28] criteria using a semi-structured face to face clinical interview (SCID-I) [33]. Additional sociodemographic-clinical information was collected including age, weight, marital status, education and occupation and clinical-psychopathological variables. As a standard procedure of clinical assessment in the ED unit of our hospital, all the participants completed the questionnaires individually and voluntarily before starting the treatment. The same assessment was repeated at the end of the treatment. Throughout the duration of the treatment, patients kept a daily food and purging diary [34]. These food diaries also collected information about daily frequency of binging, purging and exercise. The information of food diaries was used as a therapeutic tool during the treatment sessions, i.e., this information was discussed with the therapist and the rest of members of the group in every session in order to increase awareness about bulimic symptoms. Weekly binge-eating and purging frequency was determined by examining these food diaries and calculating their mean values.

The study was approved by the Ethics Committee of our institution (Ethics Committee of Clinical Research of the University Hospital of Bellvitge) and written informed consent was obtained from all participants.

### Treatment

Treatment consisted of a 22 outpatient 90-minute weekly sessions. There were a total of 8–10 patients per group. These comprised 6 initial sessions of psychoeducational brief group therapy [35] followed by 16 weekly outpatients sessions of CBT [32]. This program and its complementary material have already been manualized and published in Spanish [34] with demonstrated effectiveness [36]. BN and BED patients were placed in separated treatment groups, but both treatment groups were based on the same CBT program. Patients who completed treatment were assessed at end of CBT therapy and classified into three categories “full remission”, “partial remission” or “non-remission” group, which was based on treatment outcomes. Primary outcome was based on the food and purge diary and the response of some clinical questionnaires in the field of ED. The working definition of a “full remission” outcome required the absence of binging and purging (laxatives and/or vomiting) behaviors for at least 4 (consecutive) weeks and psychological improvement measured by clinical questionnaires. “Partial remission” was defined as substantial symptomatic improvement but still presence of residual symptoms (reduction of at least 50% of bulimic symptoms), and the patients who presented bad outcome was defined as “non-remission”.

### Statistical analysis

The statistical analysis was carried out with SPSS 20 for Windows. Logistic regressions, adjusted by age, compared the criteria (dependent variables) risk of remission (full-partial-no remission) and the risk of therapy dropout (present vs absent) between the three diagnostic subtypes. Analysis of variance procedures (ANOVA, also adjusted by the covariate age), compared the quantitative outcomes analyzed in this study between the different diagnostic conditions. Survival analyses through Cox’s regressions adjusted by age compared the time to dropout of therapy. Survival analyses involve the modeling of time to event data whereby “death” (or failure) is considered an event (in this study the register of the dropout), allowing censored values (in this study right censored data identified patients who didn’t dropout, that is, those who stayed for the entire treatment). The models adjusted with survival in this work attempts to answer the next two questions: a) what is the fraction of sample which will survive (in this study, survive is equivalent to not dropout) past a certain time? and b) of those that survive, at what rate will they present the event (fail)? The statistical procedure with survival included all the participants at the beginning, since it considers as outcome the “time to the presence of a dropout” (in the case of non dropout, survival time is defined as time of follow-up for the participant). Due to the multiple comparisons, Bonferroni-Holm’s correction was used to prevent increase in Type I error (the total alpha level was established at 0.05). This method for adjusting global α-level is included into the closed-test-procedures and it controls the family-wise error rate, operating in a more powerful way than the usual Bonferroni’s-adjustment.

## Results

### Socio-demographic characteristics and information regarding eating disorders

Data of this work correspond to a total sample of 454 eating disorder patients (327 BN-P, 40 BN-NP and 87 BED). There were statistical significant differences between BED and BN groups in several variables. A lower number of patients in the BED group was single. Patients in the BED group were also older and showed a significantly higher current, maximum and minimum Body Mass Index (BMI), developed their disorder at a later age of onset and suffered from it longer than the other two groups. See Table 1.

**Table 1:** Socio-demographic and information regarding eating disorders (n = 454)

		**Descriptives**			**Factor**		^**1**^**Contrasts: mean difference (MD) or OR**					
		**BN-P**	**BN-NP**	**BED**	**Group**		**BN-NP vs BN-P**		**BED vs BN-P**		**BED vs BN-NP**	
		**(n = 327)**	**(n = 40)**	**(n = 87)**	**F or χ**^**2**^	***p***	**MD or OR**	***p***	**MD or OR**	***p***	**MD or OR**	***p***
Body Mass Index (current); *mean (SD)*		24.3 (5.2)	26.4 (5.6)	35.9 (5.6)	134.5	<.001	2.07	.086	11.5*	.001	9.46*	001
Body Mass Index (Maximum); *mean (SD)*		27.4 (5.6)	28.0 (5.5)	37.3 (5.5)	89.04	<.001	0.61	.823	9.88*	.001	9.27*	.001
Body Mass Index (Minimum); *mean (SD)*		19.8 (2.9)	20.2 (2.7)	23.8 (3.9)	44.73	<.001	0.39	.790	3.99*	.001	3.61*	.001
Number of previous treatments; *mean (SD)*		0.72 (0.9)	0.63 (0.7)	0.48 (0.9)	2.401	.092						
Motivation to treatment	Own motivation	55.4%	60.0%	55.6%	14.95	.060						
	Derivation	26.0%	35.0%	37.0%								
	Pressures	18.6%	5.0%	7.4%								
Civil status; %	Single or separated	81.8%	80.0%	53.7%	38.48	<.001	1.12	.787	3.87*	.001	3.45*	.001
	Married-couple	18.2%	20.0%	46.3%								
Employment status; *%*	Employed	82.5%	86.1%	80.0%	0.657	.720						
Age (years-old); *mean (SD)*		26.2 (6.9)	27.2 (9.1)	34.1 (9.6)	35.27	<.001	1.04	.721	7.91*	.001	6.87*	.001
Onset of ED (years-old); *mean (SD)*		19.3 (6.3)	19.8 (8.9)	23.2 (11.0)	7.352	.001	0.45	.947	3.88*	.001	3.43*	.042
Length of ED (years); *mean (SD)*		7.5 (5.7)	7.0 (5.4)	10.6 (8.1)	7.809	<.001	0.55	.883	3.10*	.001	3.66*	.016

^1^Mean difference for quantitative variables and odds ratio for categorical, for significant comparisons. P-values in the table include Bonferroni-Holm’s correction.

### CBT treatment response and dropout rates across the BN-P, BN-NP and BED groups

For subjects who completed therapy, the percentage of patients who were considered in full or partial remission differed according to diagnoses subtypes (Table 2) with a statistically higher number of patients in the “full remission” group among the BED diagnosis when compared to BN-P (p = .004) and BN-NP (p < .001). The study found no statistically significant differences in the number of patients who remitted between BN-P and BN-NP (p = .687). The remission rates for the total sample (including the participants who drop-out, as an intent-to-treat analysis) showed similar results: the probability of full remissions were 47.1% for BED (95% CI: 36.6% to 57.6%), 30.0% for BN-NP (95% CI: 15.8% to 44.2%) and 27.2% for BN-P (95% CI: 22.4% to 32.0%). The risk of dropout also differed between groups, and post-hoc comparisons indicated that the incidence ratio was statistically equal for BN-P and BN-NP (p = .657), but BED showed higher rates of dropout than BN-P (p < .001) and BN-NP (p = .035) Table 2.

**Table 2:** Response to treatment from the three diagnostic groups

		**BN-P (n = 327)**	**BN-NP (n = 40)**	**BED (n = 87)**	**p**
*Remission (for completers)	Full	30.9%	35.5%	69.5%	<.001
	Partial	43.1%	35.3%	20.3%	
	No	26.0%	29.4%	10.2%	
*Remission (for total sample)	Full	27.2%	30.0%	47.1%	<.001
	Partial	37.9%	30.0%	13.8%	
	No	22.9%	25.0%	6.9%	
Dropout from treatment	Yes	12.8%	15.4%	33.7%	<.001
	No	87.2%	84.6%	66.3%	

*Outcome obtained for participants who completed the treatment.

P-values in the table include Bonferroni-Holm’s correction.

Comparisons between participants who dropout and non-dropout into each diagnostic condition showed no statistical differences in civil status (p = .773) and employment status (p = .069), patients’ age (p = .248), onset of eating disorder (p = .197) or evolution of eating disorder (p = .590). Mean scores of EDI-2 and SCL-90-R at baseline (Table 3) did not achieve significant results, except for the EDI-2 “Maturity fears” into BN-NP patients: dropouts obtained higher mean than non-dropouts (11.83 vs 7.28; p = .045) Table 3.

**Table 3:** Clinical comparison of drop-out and non-dropout at baseline for BN-P, BN-NP and BED patients

	**BN-P**					**BN-NP**					**BED**				
	**Mean; SD**					**Mean; SD**					**Mean; SD**				
	**Dropout: No (n = 285)**		**Dropout: Yes (n = 42)**		***p***	**Dropout: No (n = 34)**		**Dropout: Yes (n = 6)**		***p***	**Dropout: No (n = 59)**		**Dropout: Yes (n = 28)**		***p***
EDI: Drive for thinness	15.08	5.03	13.40	5.86	.055	15.25	4.53	15.50	4.14	.901	12.96	4.16	12.15	5.10	.442
EDI: Body dissatisfaction	18.51	7.28	18.40	8.04	.929	20.06	5.84	17.00	6.32	.252	21.47	5.91	23.04	5.10	.243
EDI: Interocep. awar.	13.50	6.61	13.33	5.78	.871	12.44	6.56	12.67	4.23	.935	10.82	6.09	11.11	6.30	.840
EDI: Bulimia	10.40	5.16	10.83	5.25	.628	10.22	4.56	9.17	4.49	.606	10.11	3.79	10.56	4.00	.624
EDI: Interper. distrust	6.77	4.90	6.05	4.41	.379	5.37	4.32	4.17	2.79	.516	4.78	4.05	5.04	4.70	.800
EDI: Ineffectiveness	12.31	6.79	13.15	8.37	.481	10.16	6.18	10.50	5.39	.899	11.53	6.67	10.11	6.44	.364
EDI: Maturity fears	7.83	5.78	9.45	6.19	.102	7.28	4.82	11.83	5.49	.045*	6.89	5.16	8.00	6.24	.396
EDI: Perfectionism	6.51	4.41	6.63	3.95	.871	5.56	3.63	4.50	7.04	.579	4.44	3.66	4.52	4.37	.929
EDI: Impulse regulation	7.84	6.37	9.41	6.15	.156	6.80	5.40	8.33	5.61	.532	6.11	5.22	6.70	7.26	.672
EDI: Ascetism	7.77	4.12	7.79	3.66	.968	7.93	3.88	7.83	4.75	.956	6.84	3.13	6.81	3.25	.977
EDI: Social insecurity	8.96	5.01	9.26	4.75	.735	7.17	4.27	6.00	2.37	.523	6.80	3.73	7.78	4.96	.321
EDI: Total score	116.3	41.4	116.8	40.5	.950	109.0	33.8	107.5	33.6	.921	102.8	31.5	105.8	34.1	.688
SCL: Somatization	1.91	0.97	1.96	0.83	.792	1.52	0.95	1.76	1.05	.584	1.96	0.99	1.94	0.89	.937
SCL: Obsessive-compul.	2.09	0.85	2.20	0.68	.432	1.91	0.82	2.23	1.01	.395	1.90	0.92	1.87	0.84	.874
SCL: Interpersonal sensit.	2.24	0.84	2.29	0.93	.757	1.98	0.95	1.87	0.63	.791	2.07	0.88	2.23	0.90	.431
SCL: Depressive	2.44	0.82	2.62	0.75	.199	2.12	0.92	2.14	0.83	.964	2.24	0.86	2.28	0.87	.836
SCL: Anxiety	1.94	0.88	2.07	0.89	.370	1.59	0.83	2.02	1.15	.290	1.58	0.85	1.70	0.84	.566
SCL: Hostility	1.55	0.98	1.88	1.01	.056	1.38	0.91	2.05	1.31	.129	1.29	0.91	1.54	1.01	.271
SCL: Phobic anxiety	1.26	1.00	1.32	0.93	.742	1.05	0.79	1.12	0.85	.844	.84	0.80	.95	0.84	.591
SCL: Paranoid Ideation	1.65	0.87	1.79	0.96	.354	1.25	0.79	1.61	1.00	.333	1.30	0.81	1.51	0.84	.272
SCL: Psychotic	1.45	0.74	1.63	0.70	.170	1.27	0.69	1.67	0.69	.210	1.25	0.65	1.25	0.73	.972
SCL: GSI score	1.92	0.73	2.06	0.64	.260	1.64	0.70	1.89	0.83	.441	1.73	0.67	1.84	0.67	.497
SCL: PST score	68.19	16.96	71.28	13.65	.278	62.20	18.48	69.50	14.96	.371	62.65	17.45	64.96	15.54	.567
SCL: PSDI score	2.44	0.56	2.54	0.48	.277	2.29	0.52	2.34	0.69	.818	2.43	0.43	2.50	0.52	.494

*Bold: significant comparison (.05 level). P-values in the table include Bonferroni-Holm’s correction.

Considering dropouts across diagnostic subtypes (BN-P, BN-NP and BED), no statistical differences emerged by civil status (p = .133), employment status (p = .271), onset of eating disorder (p = .167) and evolution of disease (p = .125), but patients who dropout were older in BED cohort (mean = 32.2, SD = 9.5) compared to BN-P (mean = 25.5, SD = 6.7) and BN-NP (mean = 22.8, SD = 3.7). Mean scores of EDI-2 and SCL-90-R at baseline were also statistically equal between diagnostic subtypes, with the exception of EDI-2 “Body dissatisfaction”, which achieved the higher mean for BED (23.0) compared to BN-P (18.4) and BN-NP (17.0) Table 4.

**Table 4:** Clinical comparison of dropouts at baseline

	**Mean; SD**						
	**Dropout BN-P (n = 42)**		**Dropout BN-NP (n = 6)**		**Dropout BED (n = 28)**		***p***
EDI: Drive for thinness	13.4;	5.9	15.5;	4.1	12.1;	5.1	.357
EDI: Body dissatisfaction	18.4;	8.0	17.0;	6.3	23.0;	5.1	.019*
EDI: Interoceptive awareness	13.3;	5.8	12.7;	4.2	11.1;	6.3	.324
EDI: Bulimia	10.8;	5.3	9.2;	4.5	10.6;	4.0	.730
EDI: Interpersonal distrust	6.05;	4.41	4.17;	2.79	5.04;	4.70	.487
EDI: Ineffectiveness	13.2;	8.4	10.5;	5.4	10.1;	6.4	.249
EDI: Maturity fears	9.45;	6.19	11.8;	5.49	8.00;	6.24	.344
EDI: Perfectionism	6.63;	3.95	4.50;	7.04	4.52;	4.37	.132
EDI: Impulse regulation	9.41;	6.15	8.33;	5.61	6.70;	7.26	.263
EDI: Ascetism	7.79;	3.66	7.83;	4.75	6.81;	3.25	.536
EDI: Social insecurity	9.26;	4.75	6.00;	2.37	7.78;	4.96	.197
EDI: Total score	116.8;	40.5	107.5;	33.6	105.8;	34.1	.490
SCL: Somatization	1.96;	0.83	1.76;	1.05	1.94;	0.89	.876
SCL: Obsessive-compulsive	2.20;	0.68	2.23;	1.01	1.87;	0.84	.203
SCL: Interpersonal sensitivity	2.29;	0.93	1.87;	0.63	2.23;	0.90	.572
SCL: Depressive	2.62;	0.75	2.14;	0.83	2.28;	0.87	.152
SCL: Anxiety	2.07;	0.89	2.02;	1.15	1.70;	0.84	.238
SCL: Hostility	1.88;	1.01	2.05;	1.31	1.54;	1.01	.326
SCL: Phobic anxiety	1.32;	0.93	1.12;	0.85	.95;	0.84	.248
SCL: Paranoid Ideation	1.79;	0.96	1.61;	1.00	1.51;	0.84	.485
SCL: Psychotic	1.63;	0.70	1.67;	0.69	1.25;	0.73	.093
SCL: GSI score	2.06;	0.64	1.89;	0.83	1.84;	0.67	.389
SCL: PST score	71.3;	13.6	69.5;	15.0	65.0;	15.5	.228
SCL: PSDI score	2.54;	0.48	2.34;	0.69	2.50;	0.52	.663

*Bold: significant comparison (.05 level). P-values in the table include Bonferroni-Holm’s correction.

**Figure 1:**
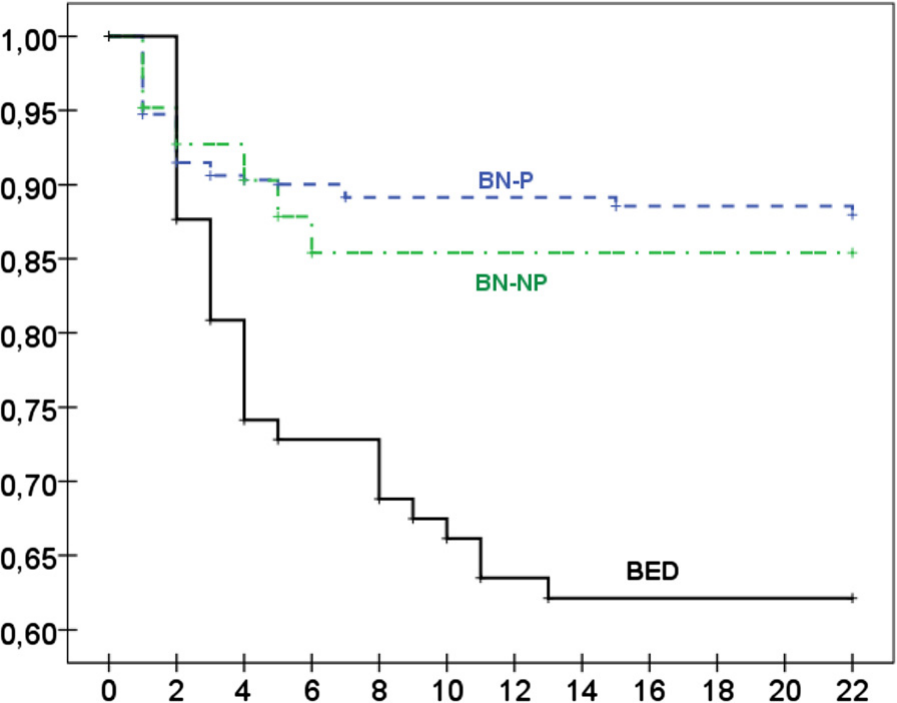
Survival function (at mean of covariate age) for the time (session) to the dropout of treatment.

Figure 1 shows the plots for the survival function (at mean of covariate age), representing in the X-axis the number of sessions to the dropout of the treatment and in the Y-axis the cumulate survival probability (%). These curves represent the probability that a patient “survives dropout-free” for at least a specific time or longer, and they can be interpreted as a measure of the rate (velocity) of dropouts in each diagnostic condition. The best clinical result (low rate of dropout) corresponded to BN-P patients, followed by BN-NP. BED patients achieved a very different result, with more relevant slopes in the survival function. For BN patients (purgative and non-purgative), the high risk of dropout corresponds to the first two sessions of therapy (approximately 8% of the patients dropped out during this time). For BED patients, 12% of participants dropped out at session 2, and this risk was clearly higher than for BN until session 13 (when the last dropout was registered). Cox’s regression adjusted by age obtained significant differences in the functions for the three diagnostic subtypes (χ^2^(Wald) = 20.78, df = 2, p < .001); The comparison of the three diagnosis showed no statistical differences between BN-P and BN-NP (p = .652), while BED differed from BN-P (p < .001; OR = 3.37, 95% CI: 1.99 to 5.71) and BN-NP (p = .027; OR = 2.77, 95% CI: 1.12 to 6.85).

#### Comparison of pre-post changes for clinical and psychopathological outcomes

Regarding BMI, no statistical differences emerged in the pre-post changes in each diagnostic subtype (p = .259). Table 5 contains the mean scores for quantitative outcomes (improvement of the psychological symptoms) before and after the treatment for each diagnosis state, as well as the ANOVA adjusted by patients’ age that valued the changes pre-post therapy (p-value) and the specific contrast for mean differences into each diagnostic subtype. BN-P achieved statistical significant pre-post changes in all the measures, except for SCL-90-R GSI index. BN-NP patients achieved significant pre-post differences in the mean scores in Drive for Thinness, Body Dissatisfaction, Bulimia and EDI-2 total score. BED patients showed significant pre-post changes in many EDI-2 scales (excluding Interpersonal Distrust, Maturity Fears and Perfectionism) and the SCL-90-R scores (except for Paranoid Ideation and GSI index).

**Table 5:** Comparison of pre-post changes for clinical outcomes between diagnostic subtypes

	**Means**						**ANOVA comparing the pre-post change**			
	**BN-P (n = 327)**		**BN-NP (n = 40)**		**BED (n = 87)**			**Φ (mean difference): pre-post**		
	**Pre**	**Post**	**Pre**	**Post**	**Pre**	**Post**	***p***	**BN-P**	**BN-NP**	**BED**
EDI: Drive for thinness	14.86	12.42	15.29	13.27	12.70	12.72	.014	2.54*	2.26*	1.90*
EDI: Body dissatisfaction	18.50	16.28	19.58	16.67	21.99	20.30	.004	1.98*	3.59*	3.28*
EDI: Interocep. awar.	13.48	10.91	12.47	10.09	10.91	8.77	.022	2.53*	2.04	2.41*
EDI: Bulimia	10.46	5.78	10.05	4.58	10.26	4.56	.004	4.51*	5.62*	5.95*
EDI: Interper. distrust	6.68	5.36	5.18	4.70	4.87	4.58	.004	1.32*	0.68	0.10
EDI: Ineffectiveness	12.42	10.24	10.21	10.21	11.06	9.86	.004	1.99*	0.13	2.89*
EDI: Maturity fears	8.04	6.78	8.00	6.58	7.26	6.86	.004	1.04*	1.05	0.42
EDI: Perfectionism	6.52	5.90	5.39	5.76	4.46	4.07	.014	0.67*	0.01	0.19
EDI: Impulse regulation	8.08	5.70	7.06	5.55	6.30	4.12	.004	2.18*	0.87	1.97*
EDI: Ascetism	7.77	6.03	7.92	7.27	6.83	5.35	.004	1.76*	0.72	1.36*
EDI: Social insecurity	9.01	7.38	6.97	7.16	7.12	5.42	.004	1.62*	−0.29	1.87*
EDI: Total score	116.4	97.4	108.8	94.9	103.8	86.6	.004	18.7*	15.2*	19.8*
SCL: Somatization	1.92	1.59	1.56	1.32	1.95	1.62	.004	0.31*	0.23	0.51*
SCL: Obsessive-compul.	2.11	1.88	1.96	1.73	1.89	1.56	.004	0.24*	0.18	0.39*
SCL: Interpersonal sensit.	2.25	1.90	1.96	1.81	2.12	1.61	.004	0.35*	0.19	0.53*
SCL: Depressive	2.47	2.05	2.12	1.91	2.25	1.79	.004	0.39*	0.24	0.54*
SCL: Anxiety	1.96	1.65	1.66	1.50	1.62	1.32	.004	0.30*	0.09	0.44*
SCL: Hostility	1.61	1.33	1.49	1.38	1.37	1.00	.020	0.19*	0.06	0.40*
SCL: Phobic anxiety	1.27	1.10	1.06	.98	.88	.68	.014	0.16*	0.06	0.30*
SCL: Paranoid Ideation	1.67	1.44	1.31	1.12	1.37	1.13	.004	0.20*	0.15	0.25
SCL: Psychotic	1.48	1.20	1.34	1.18	1.25	.92	.004	0.27*	0.11	0.49*
SCL: GSI score	1.95	1.74	1.68	1.49	1.76	1.38	.344	0.18	0.16	0.48
SCL: PST score	68.67	61.24	63.42	58.63	63.40	53.89	.004	7.25*	4.09	11.8*
SCL: PSDI score	2.46	2.25	2.30	2.12	2.45	2.06	.004	0.19*	0.17	0.45*

*Bold: significant comparison/contrast (.05). P-values include Bonferroni’s Holm correction.

## Discussion

This study aims to move the debate about bulimic disorders diagnoses, one step forward from previous studies, analyzing response to treatment in bulimic spectrum syndromes by comparing treatment outcome to group CBT between the three diagnoses subtypes (BN-P, BN-NP and BED). It aims to provide information that may be useful in the revision of the new edition of DSM (DSM-5), since an adequate diagnostic categorization requires information regarding treatment outcome.

The study confirmed previous findings [3,37], regarding socio-demographic and eating disorders characteristics with older age, a later age of onset and a longer duration of illness among the BED group of patients when compared to any of the BN subtypes.

The study demonstrated the effectiveness of CBT group therapy for the treatment of the three bulimic syndromes, both in remission (rates between 70%-90%) and in the improvement of psychological symptoms measured by clinical questionnaires (mainly improvement in Drive for Thinness, Body Dissatisfaction and Bulimia subscales), which confirms previous literature [22-25,38]. Our first hypothesis regarding treatment response among the three bulimic syndromes was only partially supported. While, following CBT group treatment, a higher number of BED patients were considered to have their symptoms remitted when compared to patients suffering from BN, no differences were found between BN-P and BN-NP in remission rates. Those findings support a previous study [17], but they are in disagreement with other studies that have shown a gradual difference in recovery from BN-P (lowest remission) through BN-NP (highest remission) [39,40].

Our second hypothesis regarding dropouts was not supported by our findings as our results indicated that the risk of dropout was statistically higher for BED and equal for purging and non-purging BN. These results are not in accordance with a previous study who found a higher rate of treatment dropout in BN than in BED [27]. However, these authors used a CD-Rom CBT treatment while we used the classical CBT outpatient treatment. Our results showed no significant differences in the clinical or psychopathological variables between BED patients who dropout vs. non-dropout, except on Body Dissatisfaction. Therefore, based on a clinical perspective, we hypothesized that the higher dropout rates in BED group could be related to the lack of weight lost while on treatment as many BED were found to be overweight or obese [22] and dieting while in treatment was not allowed. On the basis of these findings, a recent study found that CBT improves eating disorder psychopathology and psychosocial functioning in BED patients, but the lack of weight loss negatively influences the improvement profile [41]. Moreover, the higher scores on Body Dissatisfaction in BED patients who dropped out suggest that the dissatisfaction with shape and weight in these patients and the urge to lose weight may have influenced the high dropout rates.

This study is limited by the lack of information regarding psychiatric co-morbidity (mainly affective, anxiety and personality disorders) which could explain response rates and the lack of follow up data. Furthermore*,* although patients were asked whether they received previous treatment for their eating disorder (and we found no significant differences in the number of previous treatments for ED), the type of treatment was not recorded. Future studies should aim to collect this information and to replicate this study using other treatments modalities found to be effective in bulimic disorders, such as Interpersonal Psychotherapy [42,43]. Furthermore, future studies should control for pharmacotherapy during CBT, as the lack of this data is a limiting factor of the present study. In spite of these limitations, the current study has, for the first time, addressed treatment response and dropout rates of CBT group therapy across the three bulimic disorders, including a large sample of BN-NP which, to our knowledge, has not been attempted before.

## Conclusions

The results of this study reinforce the arguments of the new Diagnostic and Statistical Manual of Mental Disorders (DSM-5) to include the diagnostic category of BED as a separate category and not within the Eating Disorders Not Otherwise Specified (EDNOS). It also supports the new proposed classification to include BN-P and BN-NP in a single diagnostic category called Bulimia Nervosa, without purging subtypes.

## Abbreviations

A: Ascetism; BED: Binge eating disorder; B: Bulimia; BD: Body dissatisfaction; BMI: Body mass index; BN: Bulimia nervosa; BN-P: Bulimia nervosa-purging type; BN-NP: Bulimia nervosa-non purging type; CBT: Cognitive behavioural therapy; DSM-IV-TR: Diagnostic and statistical manual of mental disorders 4th edition revised; DSM-5: Diagnostic and statistical manual of mental disorders 5th edition; DT: Drive for thinness; ED: Eating disorders; EDI-2: Eating disorders inventory-2; EDNOS: Eating disorders not otherwise specified; GSI: Global severity index (SCL-90-R); I: Ineffectiveness; IA: Interoceptive awarness; ID: Interpersonal distrust; IR: Impulse regulation; MF: Maturity fears; P: Perfectionism; PSDI: Positive symptom distress index (SCL-90-R); PST: Positive symptom total (SCL-90-R); SCID-I: Structured clinical interview for DSM-IV-TR axis I disorders; SCL-90-R: Symptom checklist- revised; SI: Social insecurity.

## Competing interests

All authors declare that they have no conflicts of interests.

## Authors’ contributions

ZA, NR, SJM, EV, IS AND FFA designed the study. ZA, NR, EV and IS collected the patient data. RG performed the statistical analyses. ZA, EPL, JA and NR wrote the first draft of the manuscript. All authors commented on and approved the final manuscript.

## Pre-publication history

The pre-publication history for this paper can be accessed here:

http://www.biomedcentral.com/1471-244X/13/285/prepub
